# GENDER INFLUENCES ON LIFE SPACE TRAVEL AMONG THE OLD-OLD

**DOI:** 10.1093/geroni/igz038.1173

**Published:** 2019-11-08

**Authors:** Chengming Han, Eva Kahana, Boaz Kahana, Tirth Bhatta

**Affiliations:** 1 MA, Cleveland, Ohio, United States; 2 Case Western Reserve University, Cleveland, Ohio, United States; 3 Cleveland State University, Cleveland, Ohio, United States; 4 UNLV, Las Vegas, Nevada, United States

## Abstract

This paper focuses on gender influences on life space mobility based on distance from home traveled by elderly retirees. Consideration of life space travel offers a window into environmental autonomy and complexity in late life. The gendered nature of time use and social networks have been primarily studied in younger age groups. Our sample included 437 older adults (mean age 83 for both men and women) living in a large Florida retirement community that offered no services. Mean age was 83 for both men and women. Fewer (37.6%) women than men (69.7%) were married and more men drove a car (83% vs 63%).Women reported poorer subjective health and had more IADL limitations. Compared to women, men were significantly more likely to travel long distances. Women’s weekly and monthly travel tended to be local, limited to their neighborhood. On average, respondents of both genders visited their families and friends out of town every month. Better health, current driving and volunteering were related to longer driving distances for both genders, but these advantages were no longer significant after controlling for demographic characteristics. Our findings underscore the complex relationships between gender and life space travel in late life. Even among elderly men and women of the same age better health and driving resources contribute to larger life space for elderly men.

